# Noninvasive low-frequency electromagnetic stimulation of the left stellate ganglion reduces myocardial infarction-induced ventricular arrhythmia

**DOI:** 10.1038/srep30783

**Published:** 2016-07-29

**Authors:** Songyun Wang, Xiaoya Zhou, Bing Huang, Zhuo Wang, Liping Zhou, Menglong Wang, Lilei Yu, Hong Jiang

**Affiliations:** 1 Department of Cardiology, Renmin Hospital of Wuhan University, Cardiovascular Research Institute of Wuhan University, Wuhan, 430060, Hubei, China

## Abstract

Noninvasive magnetic stimulation has been widely used in autonomic disorders in the past few decades, but few studies has been done in cardiac diseases. Recently, studies showed that low-frequency electromagnetic field (LF-EMF) might suppress atrial fibrillation by mediating the cardiac autonomic nervous system. In the present study, the effect of LF-EMF stimulation of left stellate ganglion (LSG) on LSG neural activity and ventricular arrhythmia has been studied in an acute myocardium infarction canine model. It is shown that LF-EMF stimulation leads to a reduction both in the neural activity of LSG and in the incidence of ventricular arrhythmia. The obtained results suggested that inhibition of the LSG neural activity might be the causal of the reduction of ventricular arrhythmia since previous studies have shown that LSG hyperactivity may facilitate the incidence of ventricular arrhythmia. LF-EMF stimulation might be a novel noninvasive substitute for the existing implant device-based electrical stimulation or sympathectomy in the treatment of cardiac disorders.

Previous studies have demonstrated that the activation and remodeling of left stellate ganglion (LSG) induced by myocardial infarction^1^,^2^ might be the immediate triggering mechanisms of ventricular arrhythmia (VA) and sudden cardiac death^3^,^4^, and suppressing LSG neural activity might be a feasible antiarrhythmic therapy^5^. In the past decades, LSG denervation and blocking have been shown to be benefit for reducing VA^6^. However, undesirable side effects, such as cervical injury and Horner’s syndrome, have limited the clinic use of LSG denervation or blocking. Therefore, exploring a novel noninvasive approach is necessary.

Transcranial magnetic stimulation (TMS), a neurostimulation and neuromodulation technique based on the principle of electromagnetic induction of an electric field in the brain, has been proposed for treatment of a variety of neurological disorders. Previous studies has shown that TMS might mediate the cardiac rhythm by modulating the autonomic nervous system^7^. Scherlag *et al*.^8^ showed that exposure the vagal trunks or the chest to the low-frequency magnetic field (LF-EMF) might suppress atrial fibrillation, whereas exposure to the high-frequency field might induce atrial fibrillation by autonomic modulating. Recently, Yu *et al*.^9^ further demonstrated that LF-EMF stimulation of the vagal trunks or chest might suppress atrial fibrillation by inhibiting the neural activity of atrial ganglionated plexus. In this study, we hypothesized that exposure LSG to the LF-EMF might inhibit the LSG neural activity, thereby reducing VAs after acute myocardial infarction^6^.

## Results

**Figure 1:**
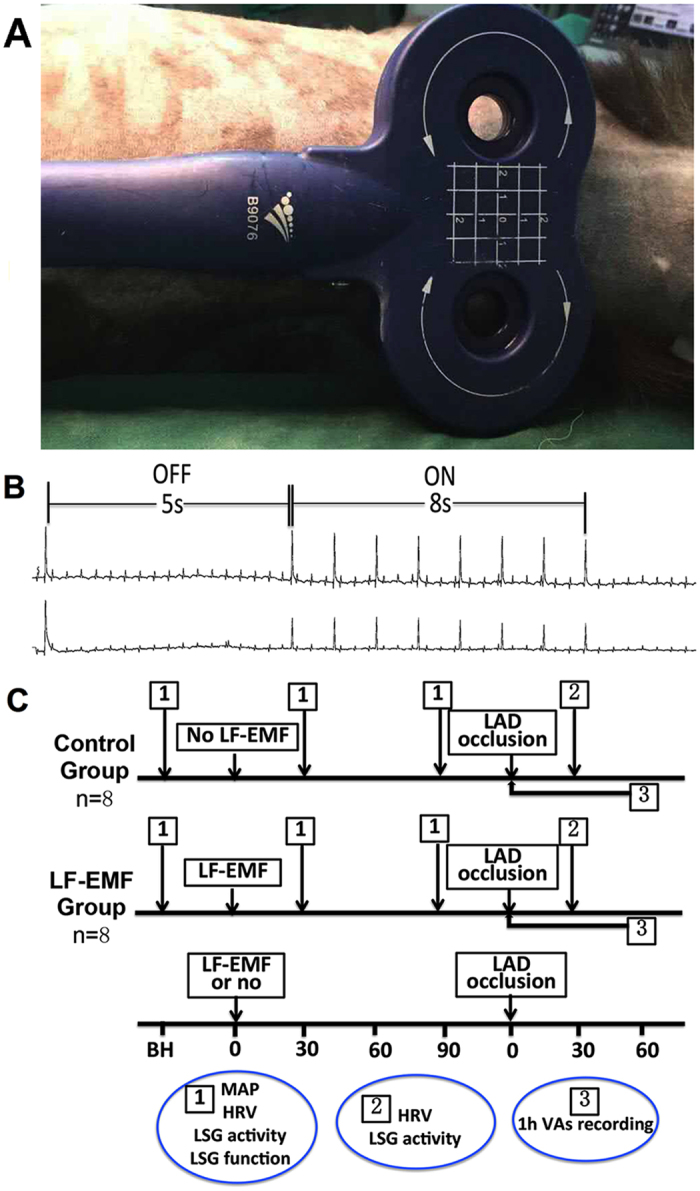
Schematic representation of the position of the LF-EMF (**A**), stimulus pattern (**B**) and the experimental design flow chart (**C**). LSG, left stellate ganglion; LF-EMF, low-frequency electromagnetic field; LAD, left anterior descending artery; MAP, monophasic action potential; HRV, heart rate variability; VA, ventricular arrhythmia.

LSG was exposed to intermittent LF-EMF stimulation before left anterior descending artery occlusion in LF-EMF group (Fig. 1A–C). Both the blood pressure and heart rate were kept at a stable level during the LF-EMF stimulation. No visible damage was shown in LSG or cardiac tissue after 90 min LF-EMF treatment. All dogs developed ECG ST-segment and/or T-wave changes acutely after ligating the left anterior descending artery.

### Effect of LF-EMF stimulation on myocardial infarction-induced VAs

**Figure 2:**
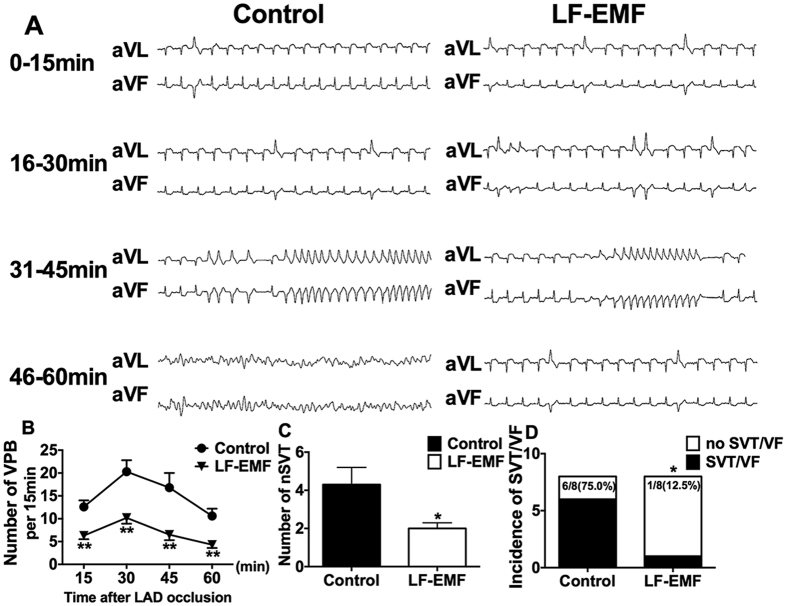
Representative examples (**A**) and the incidence (**B**–**E**) of AMI-induced VAs in the Control group (n = 8) and EMF group (n = 8). *P < 0.05 and **P < 0.05 as compared to the Control group. AMI, acute myocardial infarction; VPB, ventricular premature beats; VT, ventricular tachycardia; VF, ventricular fibrillation; other abbreviations as in Fig. 1.

Figure 2A shows the representative examples of VAs in the Control group and LF-EMF group. As compared to the Control group, both the number of ventricular premature beat (VPB) and the number of non-sustained ventricular tachycardia (VT) were significantly decreased (Fig. 2B,C). Furthermore, the incidence of sustained VT/VF was significantly suppressed (75.0% vs 12.5%, P < 0.05, Fig. 2D) in the LF-EMF group.

### Effect of LF-EMF stimulation on MAP

**Figure 3:**
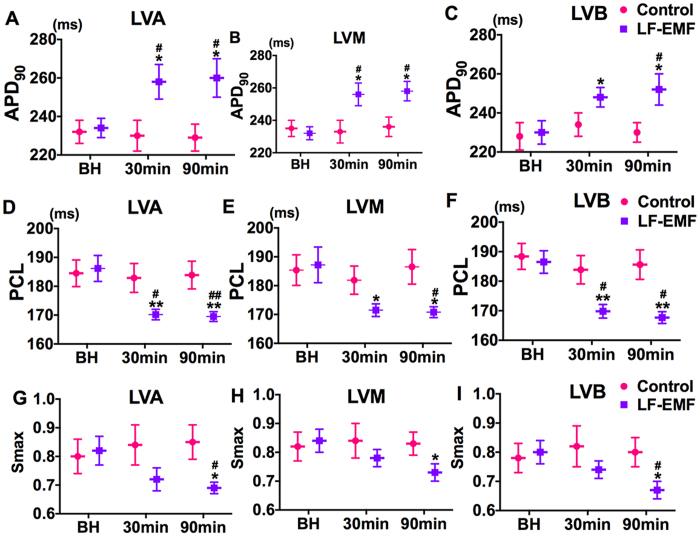
Effect of LF-EMF stimulation on APD_90_ (**A**,**B**), PCL (**C**,**D**) and Smax (**E**,**F**) in the Control group (n = 8) and EMF group (n = 8). *P < 0.05 and **P < 0.05 as compared to the group baseline; ^#^P < 0.05 and ^##^P < 0.05 as compared to the Control group. LVA, left ventricular apex; LVM, the median of left ventricle; LVB, left ventricular base; MAP, monophasic action potential; APD, action potential duration; APD_90_, monophasic action potential duration determined at 90% of repolarization; PCL, pacing cycle length of APD alternans; BH, baseline; Smax, the maximal slope of the restitution curve, other abbreviations are identical to Fig. 1.

Figure 3A–F demonstrates the effect of LF-EMF on action potential duration at 90% repolarization (APD_90_, Fig. 3A–C), pacing cycle length of action potential duration alternans (PCL, Fig. 3D–F) and the maximal slope of the restitute curve (S_max_, Fig. 3G–I). As compared to group baseline, no significant change was shown in APD_90_, PCL or S_max_ obtained from different sites of left ventricle in the Control group, whereas a significant change was shown in APD_90_, PCL and Smax of those sites both at 30 min and 90 min after LF-EMF stimulation in the LF-EMF group (Fig. 3A–F).

### Effect of LF-EMF stimulation on heart rate variability

**Figure 4:**
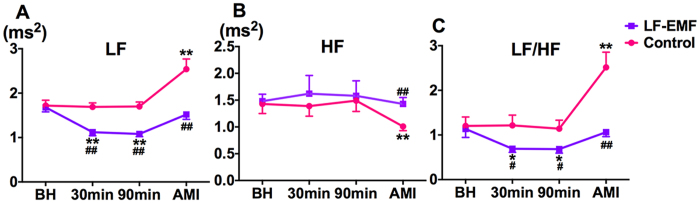
Effect of LF-EMF stimulation on LF (**A**), HF (**B**) and LF/HF (**C**) in the Control group (n = 8) and EMF group (n = 8). *P < 0.05 and **P < 0.01 vs group baseline; ^#^P < 0.05 and ^##^P < 0.05 as compared to the Control group. LF, low frequency; HF, high frequency; LF/HF, the ratio between LF and HF; BH, baseline. Other abbreviations are identical to those in Fig. 1.

Figure 4 demonstrates that both low frequency component (LF) and the ratio between LF the high component (LF/HF) were significantly decreased by LF-EMF stimulation both at 30 min and 90 min later but not by sham LF-EMF stimulation as compared to group baseline. In comparison with group baseline, acute myocardial infarction resulted in a significant change in LF (2.54 ± 0.23 ms^2^ vs 1.72 ± 0.12 ms^2^, P < 0.01, Fig. 4A), high frequency component (HF, 1.01 ± 0.08 ms^2^ vs 1.43 ± 0.18 ms^2^, P < 0.01, Fig. 4B) and LF/HF (2.51 ± 0.34 vs 1.20 ± 0.20, P < 0.01, Fig. 4C) in the Control group, whereas those were kept at a normal level in the LF-EMF group (LF, 1.52 ± 0.1 1 ms^2^ vs 1.68 ± 0.10 ms^2^; HF, 1.43 ± 0.12 ms^2^ vs 1.48 ± 0.13 ms^2^; LF/HF, 1.06 ± 0.10 vs 1.14 ± 0.19, all P > 0.05, Fig. 4A–C).

### Effect of LF-EMF stimulation on serum norepinephrine and LSG function

**Figure 5:**
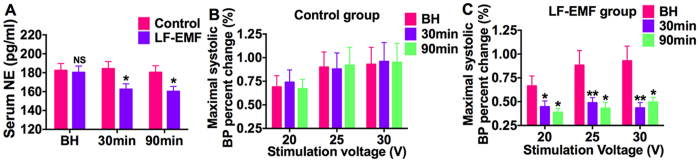
Effect of LF-EMF stimulation on serum NE (**A**) and LSG function (**B**,**C**) in the Control group (n = 8) and EMF group (n = 8). NS, P > 0.05, *P < 0.05 and **P < 0.01 as compared to the Control group at the same time point. NE, norepinephrine. Other abbreviations are alike to those in Fig. 1.

In comparison with group baseline, serum norepinephrine was decreased from 180.3 ± 6.8 pg/ml to 162.5 ± 5.8 pg/ml at 30 min later and to 160.3 ± 5.2 pg/ml at 90 min later in the LF-EMF group, whereas kept a stable level in the Control group (Fig. 5A). Furthermore, the systolic blood pressure increase in response to LSG stimulation was kept a baseline level in the Control group (Fig. 5B), whereas significantly attenuated by LF-EMF in the LF-EMF group at a voltage of 20–30 V as compared to group baseline (Fig. 5C). Take 25 V for example, the maximal systolic blood pressure increase induced by LSG stimulation was decreased from 88.3 ± 15.4% to 43.1 ± 6.2% (P < 0.01) at 90 min later, whereas kept at about 90% in the Control group (Fig. 5B,C).

### Effect of LF-EMF stimulation on the neural activity of LSG

**Figure 6:**
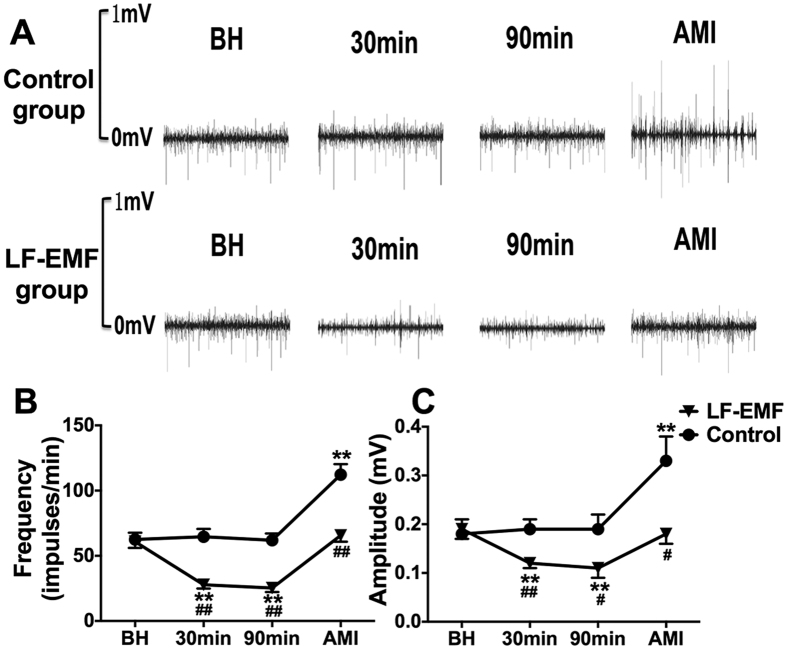
Representative examples (**A**) and quantitative analysis (**B**,**C**) of LSG neural activity in the Control group (n = 8) and EMF group (n = 8). **P < 0.01 as compared to group baseline; ^#^P < 0.05 and ^##^P < 0.05 as compared to the Control group. All abbreviations are identical to Figs 1 and 2.

Figure 6A shows the representative examples of LSG neural activity at baseline, 30 min after LF-EMF stimulation, 90 min after LF-EMF stimulation and 15 min after acute myocardial infarction. Figure 6B,C demonstrates that no significant difference was shown both in the frequency and the amplitude of LSG neural activity between the Control group and the LF-EMF group. As compared to group baseline, LF-EMF stimulation resulted in a significant decrease in LSG neural activity at 30 min and 90 min later, whereas no significant change was caused by sham LF-EMF stimulation (Fig. 6B,C). Furthermore, as compared to baseline, the neural activity was significantly increased after acute myocardial infarction in the Control group (Frequency: 62.5 ± 5.2impulse/min vs 112.2 ± 8.1impulse/min, P < 0.01; Amplitude: 0.18 ± 0.03 mV vs 0.33 ± 0.05 mV, P < 0.01) but kept at a comparable level in the LF-EMF group (Frequency: 60.8 ± 4.8impulse/min vs 65.6 ± 4.8impulse/min, P > 0.05; Amplitude: 0.19 ± 0.02 mV vs 0.18 ± 0.02 mV, P > 0.05).

## Discussion

In the present study, we applied LF-EMF at the body surface of LSG. Both the ventricular electrophysiological parameters (APD_90_, PCL, Smax) and autonomic neural activity (serum norepinephrine, LSG function and LSG neural activity) were significantly affected by LF-EMF stimulation. Furthermore, the acute myocardial infarction-induced increased neural activity of LSG was significantly attenuated and the VAs was significantly reduced by LF-EMF. These findings suggested that exposure the LSG to LF-EMF might significantly reduce the neural activity of LSG, therefore reducing the incidence of VAs.

Previous studies have shown that activation of LSG facilitates, whereas inhibition of LSG protects against VAs^4^,^10^. In the past two decades, TMS has been widely used in clinical neurology^11^,^12^. Amounts of studies have shown that high-frequency stimulation increases cortical excitability, whereas low-frequency stimulation decreases neuronal excitability^11^,^12^. Recently, studies also demonstrated that TMS might affect the cardiac rhythm by modulating the autonomic nervous system^7^. Scherlag *et al*.^8^ showed that high-frequency magnetic stimulation of the vagal nerves might induce atrial tachycardia and atrial fibrillation, which was eliminated after propranolol and atropine injection. Low-frequency stimulation of the vagal nerves, however, reduced the heart rate and decreased the voltage required to induce atrioventricular conduction block^8^. Furthermore, recent study demonstrated that exposure the heart to the LF-EMF might significantly suppress atrial fibrillation and the mechanism might be by modulating the neural activity of atrial ganglionated plexus^9^. In the present study, we found that exposure the LSG to the LF-EMF significantly reduced the serum norepinephrine, neural activity of LSG and VAs. All these indicate that noninvasive LF-EMF might reduce VAs by facilitating the autonomic rebalance, but what underlie the beneficial effects of LF-EMF on LSG was poorly defined.

In the present study, we suggested some possible mechanisms underlying the suppressing of LSG neural activity. Firstly, TMS, as an effective treatment for patients with neural disorders, has been implicated long-lasting therapeutic effects after the cessation of TMS treatment^13^. Most researchers have contributed these effects to be long-term depression (LTD) and long-term potentiation (LTP) cause the duration of the effects seemed to implicate changes in synaptic plasticity^13^. LTD is caused by low-frequency stimulation or the stimulation of a postsynaptic neuron, whereas LTP is caused by high-frequency stimulation or the stimulation of a presynaptic neuron^13^. Ca++ signal, which is known to regulate membrane excitability and modulate second messengers related to multiple receptors and signal transduction pathways, has been shown to be the major determinant whether LTD or LTD arises^14^,^15^. Recently, Scherlag *et al*.^8^ also suggested that LTP or LTD was existed cause exposure the chest to the low-frequency electromagnetic field for 35 mins might result in the suppression of atrial fibrillation for 3 to 4 hours after the application of LF-EMF. In the present study, we also found that pretreatment with LF-EMF might significantly attenuated the acute myocardial infarction-induced activation of LSG neural activity and VAs, suggesting that LTP or LTD might be a potential explain for the salutary effects of LF-EMF stimulation. Secondly, previous studies have shown that TMS might also affect the expression levels of various receptors and other neuromediators, such as β-adrenoreceptors, dopamine^11^,^16^,^17^. In the present study, serum norepinephrine was significantly decreased after exposure to the LF-EMF, indicating that modulating the neurotransmitters might be one of the underlying mechanisms underlying the salutary effects of LF-EMF stimulation. Thirdly, previous studies also showed that TMS might also modulate dentritic sprouting (axon growth) and the density of synaptic contacts, and the authors suggested that these results are associated with the Brain-derived neurotrophic factor (BDNF)-tyrosine kinase B (TrkB) signaling system^18^,^19^. BDNF, as the most abundant neurotrophin in the brain, was reported to be a major contributor to the N-methyl-D-aspartate receptor-dependent LTP and LTD processes^20^. Wang *et al*.^21^ demonstrated that low-frequency TMS might reduce BDNF levels. High-frequency stimulation, however, might increase serum BDNF levels and the affinity of BDNF for TrkB receptors. Furthermore, previous studies also showed that trancranial stimulation might result in the changes in neural-related proteins, such as c-fos and tyrosine hydroxylase, which are closely related with the neural remodeling processes^6^,^13^,^20^. Autonomic neural remodeling, however, plays a key role in the initiation and maintenance of VAs^4^,^10^. All these implicate that modulating autonomic neural remodeling might be another mechanism of the antiarrhythmic effect of LF-EMF stimulation. Fourthly, the above mainly shows the underlying mechanisms of LF-EMF stimulation, but how can the LSG perceive the LF-EMF remains unknown. During the past few decades, many mechanisms, which might provide the basis for how the animals detect magnetic fields, have been proposed^22^. However, the magnetoreceptors have not been identified with certainty in any animal, and the mode of transduction for the magnetic sense remains unknown^23^. Recently, Xie *et al*. hypothesized that the putative magnetoreceptor, the iron-sulphur cluster protein, might combine with the magnetoreception-related photoreceptor cryptochromes to form the basis of magnetoreception in animals and this was corroborated in pigeon retina^24^. Furthermore, Zhang *et al*. further showed that the cells which had been transfected iron-sulphur cluster protein might response to the remote magnetic stimulation^25^. All these indicate that the iron-sulphur cluster protein might be the potential magnetoreceptor for the animals to detect the magnetic fields.

Though the present study showed wonderful results, but there are some limitations in this study. First, anesthesia with pentobarbital might affect the autonomic nervous system. However, this could be counteracted cause anesthesia was maintained continuously during the whole surgery and conducted in a same fashion in both groups. Second, the coil used in this study is too large to achieve LSG-targeted stimulation without affecting the surrounding tissues. It would be a great step forward if the coils could be technically improved. Third, we only observed the effect of LF-EMF in acute canine model. Fourth, we mainly focused on the autonomic nervous system imbalance, one of the major contributors of post-infarction VAs, cause we intervened the LSG with LF-EMF in this study. It’s a great limitation that some other major factors, like area at risk, infarct size, degree of collateral flow and the possibility of any preconditioning pathway were not involved in this study. However, previous studies have shown that LSG activation might facilitate the incidence of VAs, whereas pre-emptive or post-ischemic/infarction LSG inhibition by blockage or denervation might decrease the incidence of VAs and improve the infarct size, collateral flow, contractile force both in animals^26^,^27^,^28^,^29^ and patients^30^,^31^. Furthermore, studies have shown that LSG stimulation might increase the likelihood of early or delayed afterdepolarization development and the initiation of reentry, thereby resulting in the incidence of VAs^32^,^33^,^34^. In this study, LF-EMF stimulation of the LSG might significantly inhibit the neural activity of LSG, thereby reducing the incidence of VAs. Therefore, it’s reasonable to refer that improving the above factors might also be the potential mechanisms underlying the beneficial effects of LF-EMF stimulation, but further studies with optimized parameters and all-round considerations are required in the future.

In conclusion, the present study showed that LF-EMF stimulation might significantly reduce the neural function and neural activity of LSG. Exposure the LSG to the LF-EMF might be a feasible method for preventing the acute myocardial infarction-induced VAs. However, larger studies with optimized parameters should be done in the chronic models to verify the beneficial effect of LF-EMF stimulation.

## Methods

### Animal preparation

Sixteen canines weighing between 20 and 25 kg were included in this study. The experiments were approved by the Animal Ethics Committee of Wuhan University under approval number 2015–0445 and followed the guidelines outlined by the Care and Use of Laboratory Animals of the National Institutes of Health. All surgeries were performed under anesthesia with sodium pentobarbital at an initial dose of 30 mg/kg and a maintenance dose of 60 mg/h. The depth of anesthesia was evaluated by monitoring corneal reflexes, jaw tone, and alterations in cardiovascular indices. The body surface electrocardiogram was recorded throughout the experiment with a computer-based Lab System (Lead 2000B, Jingjiang Inc., Wuhan, China). The core body temperature of the dogs was kept at 36.5 ± 1.5 °C. Left thoracotomy was conducted at the fourth intercostal space. At the end of the experiment, canines were a lethal dose of pentobarbital (100 mg/kg, iv).

### LF-EMF

Repeated LF-EMF was supplied by the magnetic stimulation machine (YRD CCY-I, YiRuiDe Inc., Wuhan, China) with the curve 8 coil located at the body surface of the LSG (Fig. 1A). The LSG was stimulated by intermittent (8 s ON, 10 s OFF) LF-EMF stimulation with the frequency set at 1 HZ and intensity at approximately 90% of motor threshold (Fig. 1B). Motor threshold was defined as the lowest electromagnetic intensity that induced muscle contractions in the proximal forepaw and shoulder.

### Monophasic action potential recording

Monophasic action potentials from the left ventricle were recorded with a custom-made Ag–AgCl catheter. A dynamic steady state pacing protocol (S1S1) was performed to determine action potential duration alternans^35^. The pulse train was delivered at an initial cycle length slightly shorter than the sinus cycle length and the drive train of stimuli was maintained for 30 s to ensure a steady state, and then a 2-min interruption was taken to minimize the pacing memory effects. After that, another pulse train with the PCL decreased by 10 ms was delivered until action potential duration alternans appeared. Action potential duration alternans was defined as ΔAPD_90_≥10 ms for ≥5 consecutive beats^36^. The monophasic action potential recordings were analyzed by the LEAD 2000B work station system (Lead 2000B, Jingjiang Inc. China). The APD_90_ was defined as the 90% repolarization duration and the diastolic interval was the time interval from the previous APD_90_ point to the activation time of the following beat. As described in previous studies, the dynamic action potential duration restitution curves were constructed from (Diastolic interval, APD_90_) pairs using Origin 8.0 (OriginLab, Co., Northampton, MA, USA)^35^,^37^. Slope of the shortest diastolic interval was defined as S_max_.

### Measurements of heart rate variability

Spectral power for heart rate variability was analyzed on 5-minute electrocardiogram recording segments and an autoregressive algorithm was used to analyze digitized signals from the electrocardiographic recordings. The following power spectral variables were determined: HF, LF and LF/HF^38^.

### Neural recording from the LSG

To record the neural activity of the LSG, one tungsten-coated microelectrode was inserted into the fascia of the LSG and one ground lead was connected to the chest wall. The signal of the LSG was recorded with a PowerLab data acquisition system (8/35, AD Instruments, Australia) and amplified by an amplifier (DP-304, Warner Instruments, Hamden, CT, USA). The band-pass filters were set at 300 Hz to 1 kHz and the amplification ranges from 30 to 50 times^39^. The neural activity, deflections with a signal-to-noise ratio greater than 3:1, was manually determined as described in our previous studies^39^,^40^,^41^.

### LSG function

LSG function was measured as the LSG stimulation-induced maximal change in systolic blood pressure as described in our previous study^38^. High frequency stimulation (20 Hz, 0.1 ms pulse duration) was applied to the LSG using a stimulator (Grass-S88; Astro-Med, West Warwick, RI, USA). The voltage ranged from 20 V to 30 V and increased by 5 V. To eliminate the residual effect of the LSG stimulation, each stimulation should be less than 30 s and the next stimulation should be not be taken until the blood pressure returned to a normal level.

### Blood sampling

Venous blood samples were collected. Serum was separated by centrifuging at 3000 rpm for 15 min at 4 °C, and stored at −80 °C until assayed. The serum norepinephrine level was measured with a canine-specific high sensitivity ELISA kit (Nanjing Jiancheng Bioengineering Institute, Nanjing City, China)^38^.

### Measurement of the acute myocardial infarction-induced VAs

The left anterior descending coronary artery was ligated at approximately 2.5 centimeters away from its origin to induce acute myocardial infarction. The incidence and duration of the VAs induced by acute myocardial infarction during the first hour was analyzed. The VAs recorded on the ECG were defined as following^42^: VPBs, identifiable premature QRS complexes; VT, three or more consecutive VPBs; non-sustained VT, VT terminating spontaneously within 30 s; sustained VT, VT sustained for more than 30 s; and VF, a tachycardia with random ECG morphology and an associated loss of arterial blood pressure that degenerates into ventricular asystole.

### Experimental protocol

Sixteen dogs were randomly divided into LF-EMF group (n = 8, with LF-EMF) and Control group (n = 8, with sham LF-EMF). LF-EMF (1 HZ; stimulation time 8 s; interstimulus interval, 5 s) was delivered to the surface area of LSG for 90 minutes. As shown in Fig. 1C, monophasic action potential, heart rate variability, serum norepinephrine, LSG function and LSG neural activity were measured at baseline, 30 min and 90 min after LF-EMF treatment. Measurements of heart rate variability and LSG neural activity were repeated at 15 min after acute myocardial infarction. Furthermore, the incidence of VAs was recorded during the first hour after acute myocardial infarction.

### Statistical analysis

Continuous variables are presented as the mean ± SEM and were analyzed by t test, one-way ANOVA, or two-way repeated-measures ANOVA with a Bonferroni posthoc test. To compare the incidence of VF between groups, Fisher’s exact test was used. All data was analyzed by GraphPad Prism version 5.0 software (GraphPad Software, Inc., San Diego, CA), and two-tailed P ≤ 0.05 was considered significant.

## Additional Information

**How to cite this article**: Wang, S. *et al*. Noninvasive low-frequency electromagnetic stimulation of the left stellate ganglion reduces myocardial infarction-induced ventricular arrhythmia. *Sci. Rep.* **6**, 30783; doi: 10.1038/srep30783 (2016).
